# Combination use of platelets and recombinant activated factor VII for increased hemostasis during acute type a dissection operations

**DOI:** 10.1186/s13019-014-0156-y

**Published:** 2014-09-02

**Authors:** Wen Yan, Chengluan Xuan, Guojia Ma, Liang Zhang, Ning Dong, Zijian Wang, Rihao Xu

**Affiliations:** Department of Cardiovascular Surgery, Second Hospital of Jilin University, Jilin University, Changchun, 130041 Jilin, China; Department of Anesthesiology, First Medical Hospital of Jilin University, Changchun, 130021 Jilin, China; Department of Plant Pathology, North Dakota State University, Fargo, 58102 ND USA; The emergency department of the No.1 hospital of Jilin University, Jilin University, Changchun, 130021 Jilin, China; Internal Medicine, Xiamen174 Hospital, Xiamen, 361000 Fujian China

**Keywords:** Platelets, Recombinant activated factor VII (rFVIIa), Acute type A dissection, Hemostatic strategy

## Abstract

**Background:**

Refractory blood loss is a common problem in surgeries for acute type A aortic dissections. Significant evidence has supported the benefit of using recombinant activated factor VII (rFVIIa) to control of intractable bleeding in patients after cardiac surgery. In this prospective clinical study, we present a novel method to achieve intraoperative hemostasis by using a combination of platelets and rFVIIa during operations for acute type A aortic dissections.

**Methods:**

Between May 2009 and August 2012, 71 patients with acute type A dissections who underwent emergency surgery were prospectively included and allocated to one of the following two intervention groups for hemostasis: 3 units platelets combined with 2.4 mg rFVIIa (n = 25), and conventional methods (n = 46).

**Results:**

The patients who received the combination of platelets and rFVIIa required fewer transfusions of red blood cells (6.2 ± 3.1 units vs 9.8 ± 2.8 units; *p* < 0.05), fresh frozen plasma (736.9 ± 178.3 ml vs 1264.3 ± 245.2 ml, *p* < 0.05), platelet concentrates (3 units vs 5.0 ± 1.8 units, *p* < 0.001), and cryoprecipitate (2.8 ± 0.9 units vs 8.2 ± 2.3 units, *p* < 0.05). These patients also required less time for sternal closure (76.9 ± 17.2 min vs 102.3 ± 10.7 min, *p* < 0.05) compared with the conventional therapy patients. There was no statistically significant difference in the incidence of serious adverse events between these two groups.

**Conclusions:**

Using a combination of platelets and rFVIIa is an effective strategy for achieving hemostasis during acute type A dissection surgery. This hemostatic strategy does not appear to be associated with an increase in postoperative adverse events.

**Electronic supplementary material:**

The online version of this article (doi:10.1186/s13019-014-0156-y) contains supplementary material, which is available to authorized users.

## Background

An acute type A aortic dissection is the most life-threatening vascular emergency recognized to date. Since this condition is associated with an 80% mortality rate, every acute type A dissection requires emergency surgical intervention to prevent aortic rupture and death [1]. One of the most serious problems during this surgery is massive refractory blood loss. During surgery for a type A dissection, the need for a complicated aortic anastomosis; prolonged cardiopulmonary bypass (CPB) time; and, particularly, deep hypothermic circulatory arrest can induce platelet disorders, thereby increasing blood loss, increases the duration of the operation and results in an increased requirement for transfusion of allogeneic blood products. The conventional method for achieving hemostasis includes the transfusion of fresh frozen plasma, platelets, fibrinogen, and antithrombin, with the aim of normalizing coagulation, while increasing platelet count and function [2].

Recombinant activated factor VII (rFVIIa; NovoSeven, Novo Nordisk, Bagsvaerd, Denmark) is a novel hemostatic agent, which has been proven effective for the management of hemorrhage after cardiac surgery [3]-[5]. Although multiple studies have supported the efficacy and safety of rFVIIa in cardiac operations [2]-[7], there are concerns regarding the optimal dosage of rFVIIa and the timing of rFVIIa administration [6],[7]. There has not been any evidence presented regarding a standardized protocol for using rFVIIa during surgery for an acute type A dissection.

The optimal dosages of rFVIIa, as described in the literature, range from 11 to 100 μg/kg, and Gelsomino et al. [4] presented the effectiveness of a 1.2-mg rFVIIa dose in 40 patients with persistent blood loss.

We considered the function of a platelet in a hemostatic procedure and tried to develop a hemostatic strategy that used a combination of platelets and rFVIIa [6]-[9]. After preliminary exploratory management, we selected a fixed dose of platelets and rFVIIa for intraoperative use in acute type A dissection surgery. In this study, we will present the efficacy and safety of this strategy in comparison with conventional hemostatic methods.

## Methods

### Patients and data collection

In the period from May 2009 to August 2012, 71 patients with acute type A aortic dissections who underwent emergency surgery at our institution (The Second Hospital of Jilin University, Changchun, China) were prospectively included in this non-randomized clinical study. The study protocol was approved by the Ethics Committee of The Second Hospital of Jilin University. We introduced in details about the conventional hemostatic treatment and this new strategy, and made a clear comparison between them. We had made it clear and clarified to patients and their families, and they should be capable to understand the two different methods and make their decision accordingly. They made their own decision preoperatively about which hemostatic strategy would be used during operation. Of these patients, 25 (35.2%) received the combination of platelets and rFVIIa after cessation of CPB, and the others received conventional hemostatic treatment. All patient data were recorded according to a standardized procedure established by our scientific committee. Data collected included patient demographics, mortality, adverse events, and the use of blood products. In addition, hematologic data such as the international normalized ratio (INR) and activated partial thromboplastin time (APTT) were collected. The amount of time required for sternal closure was determined by the difficulty in achieving hemostasis after separation from CPB, so we chose this measurement to evaluate this new hemostatic strategy.

### Guidelines for the combination use of platelets and rfviia during surgery

Acute type A dissection operations were performed as previously described [10],[11]. The platelet protective agent tranexamic acid was bolus infused at 10 mg/kg at the beginning of surgery, and another same dosage was given after the protamine infusion in both two groups. In brief, all the patients underwent median sternotomy, femoral artery cannulation, and total CPB. After CPB was established, cooling was initiated. Cardiac arrest was accomplished by using cold cardioplegic solution after clamping of the ascending aorta. For surgery that involved hemiarch or total arch reconstruction, patients were cooled to 20°C for a period of deep hypothermia. All the patients were underwent Bentall or ascending replacement. Hemiarch replacement or total arch replacement was performed according to the condition of the arch after the tear. The stent was implanted into the distal aorta as the stented elephant trunk. In the control group, conventional methods of achieving hemostasis were performed after separation from CPB, which included the administration of red blood cells, fresh frozen plasma, platelets, and cryoprecipitate. In the platelet and rFVIIa combination group, 3 units of platelets were quickly transfused after separation from CPB, and then 2.4 mg (25.8 μg/kg-53.3 μg/kg) of rFVIIa was given by the anesthetist. If adequate levels of hemostasis were not achieved after platelet and rFVIIa administration, conventional methods for achieving hemostasis, other than platelet transfusion, which was performed only after the operation was completed, were used.

### Statistical analysis

All comparisons were performed between patients who received conventional hemostatic therapy (control group) and patients who received a combination of platelets and rFVIIa (platelet and rFVIIa group). Values were presented as mean ± standard deviation, median ± 25^th^-75^th^ percentile interquartile range, or percentages. Baseline patient characteristics (including age, weight, gender, body surface area, baseline laboratory values, American Society of Anesthesiologists' class, principal procedure performed, use of deep hypothermic circulatory arrest, CPB time, nadir hemoglobin levels during bypass, intraoperative perfusion, and sternal closure time) were compared between the two groups using the Χ^2^ test, Kruskal-Wallis test, and *t*-test. Statistical significance was determined if the *p* value was less than 0.05. Calculations were performed using the SPSS software, version 19.0 (IBM, New York, USA).

## Results

### Patient characteristics

Table 1 highlights the demographic and clinical characteristics of the 2 groups of patients. The distribution of age, gender, height, weight, body surface area, baseline laboratory values, use of deep hypothermia circulation arrest, and CPB time was provided in Table 1. There were no statistically significant differences between the control and the platelet and rFVIIa group.

**Table 1:** **Demographic features of patients**

Variable	Control (n = 46)	rFVIIa + platelet (n = 25)	***p***Value
Age (years)	52.8 ± 10.3	53.7 ± 11.3	0.724
Male sex	31 (67%)	15 (60%)	0.607
Height (cm)	168.9 ± 8.3	167.3 ± 8.6	0.449
Weight (kg)	69.7 ± 11.8	71.4 ± 11.5	0.587
Body surface area (m^2^)	1.75 ± 0.16	1.78 ± 0.19	0.647
Baseline laboratory values			
Hemoglobin (g/L)	130 ± 16	130 ± 21	0.979
Platelets counts (10^9^cells/L)	172 ± 70	161 ± 53	0.499
International normalized ratio (INR)	1.00 ± 0.10	0.98 ± 0.09	0.305
Activated partial thromboplastin time (APTT) (sec)	26.6 ± 2.1	28.6 ± 1.0	0.964
Prothrombin time (PT) (sec)	11.9 ± 0.2	11.6 ± 0.2	0.368
D-dimer (ng/ml)	2147.8 ± 512.3	2497.5 ± 532.1	0.642
Creatinine (μmol/L)	83.1 ± 20.6	87.1 ± 32.7	0.554
ASA class			
III	3(6.5%)	1(4.0%)	1
IV	43(93.5%)	24(96.0%)	1
Principal procedure			
Bental	2(4.3%)	0(0%)	0.416
Ascending aortic replacement	3(6.5%)	2(8%)	1
Bental + hemiarch replacement	1(2.2%)	1(4%)	0.584
Ascending aortic + total arch replacement	3(6.5%)	0(0%)	0.492
Bental + total arch replacement + stent implanted	3(6.5%)	1(4%)	1
Ascending aortic + total arch replacement + stent implanted	34(73.9%)	21(84.0%)	0.331
Deep hypothermic circulatory arrest time (min)	49.5 ± 14.6	50.8 ± 11.4	0.711
Selective cerebralperfusion time (min)	43.8 ± 13.2	42.7 ± 11.1	0.724
Selective cerebralperfusion flow rate (ml/min)	492.2 ± 88.3	484.8 ± 93.6	0.743
Cardiopulmonary bypass time (min)	196.3 ± 41.8	191.4 ± 26.7	0.605
Nadir hemoglobin on bypass (g/dL)	7.3 ± 1.2	7.6 ± 0.9	0.267

Values expressed as average (standard deviation) or number (percent). ASA = American Society of Anesthesiologists.

Intraoperative transfusions and the effect of the combination of platelets and rFVIIa.

**Figure 1:**
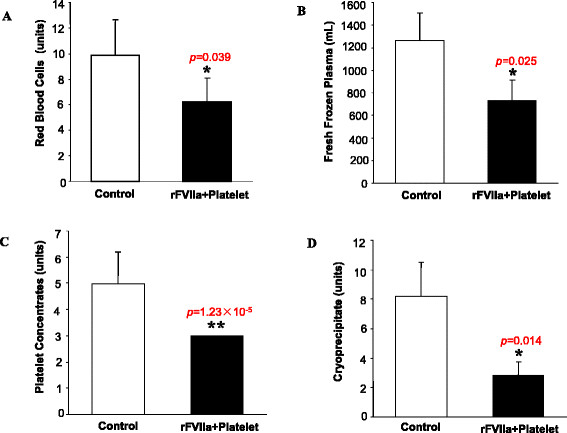
**Intraoperative blood product transfusions: A, B, C, and D bar graphs show the transfusions of red blood cells, fresh frozen plasma, platelet concentrate, and cryoprecipitate, respectively.** Data are mean ± SD (n = 46 in the control group; n = 25 in the rFVIIa + platelet group). *p < 0.05 vs control group.

**Figure 2:**
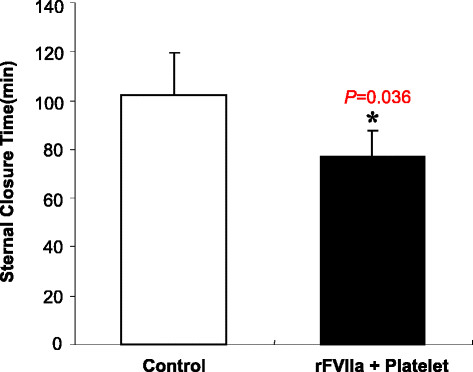
**Sternal closure time: The bar graph represents the sternal closure time.** Data are mean ± SD (n = 46 in the control group; n = 25 in the rFVIIa + platelet group). *p < 0.05 vs control group.

The patients who received the combination of platelets and rFVIIa required fewer transfusions of red blood cells (6.2 ± 3.1 units vs 9.8 ± 2.8 units; *p* < 0.05), fresh frozen plasma (736.9 ± 178.3 ml vs 1264.3 ± 245.2 ml, *p* < 0.05), platelet concentrate (3.0 units vs 5.0 ± 1.8 units, *p* < 0.001), and cryoprecipitate (2.8 ± 0.9 units vs 8.2 ± 2.3 units, *p* < 0.05) compared with the control group (Figure 1). The sternal closure time was less in the platelet and rFVIIa group as compared to the control group after separation from CPB (76.9 ± 17.2 min vs 102.3 ± 10.7 min, *p* < 0.05), which indicates that this new strategy achieves better hemostasis than conventional hemostatic methods (Figure 2).

### Coagulation laboratory findings

On arrival in the ICU, the patients in the platelet and rFVIIa group had better coagulation indicators (INR, 0.91 ± 0.11 vs 1.26 ± 0.21, *p* < 0.05; APTT, 31.6 ± 2.4 seconds vs 39.8 ± 3.7 seconds, *p* < 0.05) than did the control group patients (Table 2). Both groups had similar body temperature, and pH, hemoglobin, platelet, and fibrinogen levels.

**Table 2:** **Postoperative laboratory data**

Variable	Control (n = 46)	rFVII + platelet (n = 25)	***p***Value
Temperature (°C)	35.9 ± 0.7	35.9 ± 0.5	0.881
pH	7.41 ± 0.13	7.41 ± 0.13	0.573
Hemoglobin (g/L)	99.8 ± 12.7	104.2 ± 17.5	0.246
Platelets (10^9^cells/L)	197.4 ± 73.3	184.1 ± 77.4	0.492
International normalized ratio (INR)	1.26 ± 0.21	0.91 ± 0.11	0.008
Activated partial thromboplastin time (sec)	39.8 ± 3.7	31.6 ± 2.4	0.019
Fibrinogen (g/L)	3.02 ± 0.45	2.87 ± 0.32	0.231

Values are expressed as average (standard deviation).

### Postoperative blood loss and transfusion requirements

The amount of blood loss was similar in the 2 groups at 1, 6, and 12 h after the operation (Table 3). Patients who received the combination of platelets and rFVIIa required fewer platelet concentrate transfusions (0 unit) as compared to the control group patients on the first postoperative day (median, 1 unit; interquartile range, 0-1 unit, *p* < 0.05) and over the entire postoperative recovery period (median, 1 unit; interquartile range, 0-1 unit, *p* < 0.001; Table 3).

**Table 3:** **Postoperative bleeding and transfusions**

Variable	Control (n = 46)	rFVII + Platelet (n = 25)	***p***Value
1-hour chest tube drainage (ml)	63.6 ± 29.7	52.5 ± 31.5	0.204
6-hour chest tube drainage (ml)	229.5 ± 150.1	157.5 ± 94.2	0.227
12-hour chest tube drainage (ml)	336.3 ± 189.1	242.3 ± 132.1	0.179
Transfusions: postoperative day 0			
Red blood cell (units)	3(2-4)	2(0-4)	0.145
Fresh frozen plasma (ml)	600(300-820)	350(0-800)	0.171
Platelet concentrate (units)	1(0-1)	0	0.020
Cryoprecipitate (units)	0	0	0.180
Transfusions: Total postoperative			
Red blood cell (units)	3(2-5.5)	2(0-4)	0.246
Fresh frozen plasma (ml)	700(500-1400)	650(400-1500)	0.168
Platelet concentrate (units)	1(0-1)	0	P < 0.001
Cryoprecipitate (units)	0	0	0.180

Values are expressed as average (standard deviation) or median (interquartile range).

### Serious adverse events

Total postoperative complications were equivalent between the 2 groups (Table 4). One patient (2.2%) in the control group required reoperation on the first day after surgery for postoperative bleeding. Three patients (6.5%) in the control group and 2 patients (8%) in the platelet and rFVIIa group developed arrhythmias in the ICU, which were successfully corrected by administering the appropriate therapy. There were 2 patients (4.3%) in the control group and 1 patient (4%) in the platelet and rFVIIa group who required continuous renal replacement therapy for acute renal failure. There was 1 case of stroke each in the control group (2.2%) and in the platelet and rFVIIa group (4%), both caused by cerebrovascular ischemic abnormalities. Two patients (4.3%) in the control group died of renal failure, and 1 patient (4%) in the platelet and rFVIIa group died of stroke. The complications were not investigated and did not appear to be caused by thromboembolism or rFVIIa administration.

**Table 4:** **Adverse events**

Complication	Control (n = 46)	rFVII + platelet (n = 25)	***p***Value
Reoperation for bleeding	1(2.2%)	0	0.648
Delayed sternal closure	0	0	1
Sternal infection	0	0	1
Postoperative arrhythmia	3(6.5%)	2(8%)	0.581
Acute renal failure	2(4.3%)	1(4%)	0.718
Myocardial infarction	0	0	1
Pulmonary embolism	0	0	1
Mesenteric ischemia	0	0	1
Any stroke	1(2.2%)	1(4%)	0.584
Embolic stroke	0	0	1
Permanent paraparesis	1(2.2%)	0	0.648
Over 7 days stay in ICU	5(10.9%)	2(8%)	0.526
30-day/in-hospital death	2(4.3%)	1(4%)	0.718

Values are expressed as number (percent).

### Follow-up

There were three deaths during a mean follow-up of 33 ± 16 months in control group. One patient died of unknown cause 18 months after surgery. One patient who underwent Bental and total arch replacement combined with stent implanted received thoracoabdominal aortic replacement operation 3 years after primary surgery, and died of renal function failure. One patient died of acute myocardial infarction 2 years after surgery. There was one death during a mean follow-up of 30 ± 14 months in platelet and rFVIIa group, who died of liver cancer 3 years after surgery. The platelet and rFVIIa group had similar death rate as control group during this short follow-up (3 (6.8%) vs 1 (4.2%), *p* > 0.05)).

## Discussion

In this report, we have presented the results of administering a combination of platelets and rFVIIa during surgery for acute type A aortic dissections; this new strategy is intended to improve coagulation after separation from CPB, resulting in a reduced need for blood product transfusions and shorter sternal closure time. Our findings demonstrated that the administration of platelet supplements first, followed by a low dose of intravenous rFVIIa, significantly improved hemostasis, with few serious adverse events and no obvious increase in adverse events as compared to conventional hemostatic therapy.

Acute type A aortic dissection is the most lethal cardiovascular emergency, and uncontrolled hemorrhage is a serious, most often fatal, problem in acute type A dissection surgery. Activated factor VII is a naturally occurring initiator of hemostasis, and administration of rFVIIa has become a new tool in the management of critical hemorrhage associated with cardiovascular surgery. Many studies have reportedly proven that the rFVIIa is a safe and effective treatment for haemorrhage after cardiac surgery, but the dosage selection is still a controversy [7],[12],[13]. There have been many previous reports on the appropriate dose of rFVIIa in cardiac surgery, and the use of a low dose has been accepted in most institutions [14],[15]. We tried to establish a procedure to control hemorrhage after separation from CPB that involves the use of a low dose of rFVIIa. In our previous clinical experience with hemostasis, we found that platelet supplementation could improve the outcome of a single administration of rFVIIa. Before this strategy formation, we infused 1 unit of platelets and 1.2 mg of rFVIIa after separation from CPB, but we also needed to transfuse similar count of platelets and other blood products as before. Two units of platelet transfusion could significantly decrease hemorrhage when we infused 1 unit of platelets and 2.4 mg of rFVIIa, and it happened more than once. It is because of this that we designed a new hemostatic strategy that uses a combination of three units of platelets and 2.4 mg of rFVIIa to optimize hemostatic effects. After using this new strategy, we did not need to administer either additional platelets or rFVIIa, and we could obtain satisfactory hemostasis for sternal closure. When this study was designed, we also wanted to compare only platelet therapy and combination of platelet and rFVIIa therapy. However, during the operation process, when the only platelet therapy did not have satisfactory haemostatic results, some fresh frozen plasma and cryoprecipitate had to be used since we did not have unlimited access of platelet in our country. In the combination of platelet and rFVIIa therapy group, we also needed to use some fresh frozen plasma and cryoprecipitate. We had to try our best to get the best haemostatic result and finish the surgery as soon as possible to decrease surgical complications in both two groups.

Multiple complications related to hemostasis such as platelet dysfunction, generalized coagulation factor deficiency, and fibrinolysis can develop after acute type A dissection surgery and can contribute to coagulopathic bleeding. All of these hemostatic complications may be caused by prolonged duration on CPB and in deep hypothermia circulation arrest [7],[16],[17]. As a reportedly effective method for organ function protection during acute type A aortic dissection surgery, the deep hypothermia was extensively used during the surgery [11]. Tissue factor is an integral membrane protein that is a promoter of the coagulation cascade. The widely accepted consensus on coagulation and thrombosis is that with injury, vascular wall tissue factor is exposed to flowing blood and it forms a complex with factor VII/VIIa. Arterial thrombosis and the proliferation of thrombi require that a sequence of coagulation reactions as well as platelet deposition occur on the thrombus surface. Adding pharmacologic concentrations of rFVIIa to hemophilic blood markedly increases platelet activation in the absence of tissue factor and decreases the tissue factor-independent APTT and tissue factor-relative prothrombin time [16],[18],[19]. Therefore, the administration of a combination of additional platelets and rFVIIa could significantly improve coagulation reactions, better than a single administration of either platelets or rFVIIa alone. We obtained the corresponding conclusion from this study, in which we provide a new protocol for hemostasis.

The most important unresolved problem in hemostasis is whether rFVIIa is safe. After aortic surgery with CPB and deep hypothermia arrest, tissue factor expression is upregulated both systemically and in the areas of tissue injury [20]. Since the mechanism of rFVIIa involves binding to tissue factor, increased tissue factor expression could lead to more local and systemic thrombus formation [21]. Multiple studies have reported the safe use of rFVIIa in adults undergoing cardiac operations, but the lack of control patients in most of the studies makes it difficult to determine whether the adverse events are related to the use of rFVIIa or to the high-risk, unstable condition of patients when they receive rFVIIa. Few studies have suggested that the patients who received rFVIIa could have an increase in the number of serious adverse events, including stroke, length of hospital stay, and mortality [17],[22],[23]. A review of adverse events reported to the U.S. Food and Drug Administration suggests that most thromboembolic events, with serious morbidity and mortality, are related to the use of rFVIIa for "off-label" indications [24]. In our study, overall rates of adverse events were low and were equivalent in the treatment and control groups. Low-dose rFVIIa administration is recommended by some reports, which found that the use of lower rFVIIa doses was much safer. Using the lowest possible dose is warranted not only because of the expense of factor rFVIIa, but also because of the potential thromboembolic events [25]. The dose selected in our study (25.8 μg/kg-53.3 μg/kg) is as low as Nicholas D reported, and this dosage is safe and effective without any thromboembolic adverse events [15]. As a kind of component of blood, platelet is safe to be transfused to patient in the same blood type. However, whether the over dosed platelet transfusion would induce thromboembolic or other relevant adverse events still need to be identified in our future study. Although the cost-effectiveness outcome was not the primary aim of the current study, we did noticed that the cost of rFactor VIIa and blood products in treatment group was more than the cost of blood products in control group ($4145.9 ± 355.7 vs $3114.7 ± 847.5, p < 0.05), because the cost of rFactor VIIa at this stage is relatively high. Although the cost of rFactor VIIa could not be offset by the decreased use of blood products, our study remain of significance in that considerable blood products could be saved following the administration of platelet and rFactor VIIa. Although the overall cost may be high in the treatment, the saved blood products are definitely of value to the treatment of many other clinical conditions, such as acute hemorrhage. The blood products supply is always not enough, even seriously lack in some area, so this new strategy has important positive effect in countries and/or areas that are lack of some blood products.

The major limitation of this study is the small sample size and the lack of mechanistic research to determine the dosages of platelets and rFVIIa selected. This new hemostatic strategy was designed by using clinical experience, and it lacks laboratory study support. In addition, we did not include the data regarding the incidence of heparin induced thrombocytopenia and thrombosis (HITT) in our analyses; therefore, the incidences of HITT in both groups were unavailable. Moreover, we could not confirm the causes of the 2 cases of ischemic stroke (1 in each group). Judged from the clinical manifestation of the 2 patients and based on our experiences, HITT may be a possible reason for the stroke, and the impacts of cerebral hypo-perfusion and congenital cerebral malformation could also not be ignored. Despite of these limitations, this study is the first report of hemostatic strategy with confirmed transfusion guidelines, uniform practice, and administration of a low dose of rFVIIa.

## Conclusions

This prospective report describes a novel hemostatic strategy for the administration of a combination of platelets and rFVIIa during operations for acute type A aortic dissections, which is intended to improve intraoperative hemostasis, reduce the need for blood product transfusions, and shorten sternal closure time without a significant increase in adverse events.

## Authors’ original submitted files for images

Below are the links to the authors’ original submitted files for images. Authors’ original file for figure 1 Authors’ original file for figure 2

## Abbreviations

RFVIIa: Recombinant activated factor VII
CPB: Cardiopulmonary bypass
INR: International normalized ratio
APTT: Activated partial thromboplastin time
ICU: Intensive care unit

## References

[1] Ramanath VS, Oh JK, Sundt TM, Eagle KA (2009). Acute aortic syndromes and thoracic aortic aneurysm. Mayo Clin Proc.

[2] Von Heymann C, Redlich U, Jain U, Kastrup M, Schroeder T, Grosse J, Ziemer S, Koscielny J, Konertz WF, Wernecke KD, Spies C (2005). Recombinant activated factor VII for refractory bleeding after cardiac surgery- A retrospective analysis of safety and efficacy. Crit Care Med.

[3] Tritapepe L, De Santis V, Vitale D, Nencini C, Pellegrini F, Landoni G, Toscano F, Miraldi F, Pietropaoli P (2007). Recombinant activated factor VII for refractory bleeding after acute aortic dissection surgery: a propensity score analysis. Crit Care Med.

[4] Gelsomino S, Lorusso R, Romagnoli S, Bevilacqua S, De Cicco G, Bille G, Gensini GF (2008). Treatment of refractory bleeding after cardiac operations with low-dose recombinant activated factor VII (NovoSeven): a propensity score analysis. Eur J Cardiothorac Sur.

[5] Masud F, Bostan F, Chi E, Pass SE, Samir H, Stuebing K, Liebl MG (2009). Recombinant factor VIIa treatment of severe bleeding in cardiac surgery patients: a retrospective analysis of dosing, efficacy, and safety outcomes. J Cardiothorac Vasc Anesth.

[6] Levi M, Peters M, Buller HR (2005). Efficacy and safety of recombinant factor VIIa for treatment of severe bleeding: a systemic review. Crit Care Med.

[7] Warren O, Mandal K, Hadjianastassiou V, Knowlton L, Panesar S, John KJ (2007). Recombinant activated factor VII in cardiac surgery: a systematic review. Ann Thorac Surg.

[8] Duran M, Gunebakmaz O, Uysal OK, Ocak A, Yilmaz Y, Arinc H, Eryol NK, Ergin A, Kaya MG (2013). The relation between mean platelet volume and coronary collateral vessels in patients with acute coronary syndromes. J Cardiol.

[9] Balasubramanian V, Grabowski E, Bini A, Nemerson Y (2002). Platelets, circulating tissue factor, and fibrin colocalize in ex vivo thrombi: real-time fluorescence images of thrombus formation and propagation under defined flow conditions. Blood.

[10] Lizhong Sun MD, RuiDong Qi MD, Qian Chang MD, JunMing Zhu MD, YongMin Liu MD, ChunTao Yu MD, HaiTao Zhang MD, Bin Lv MD, Jun Zheng MD, Liangxin Tian MD, JinGuo Lu MD (2009). Surgery for acute a dissection with the tear in the descending Aorta using a stented elephant trunk procedure. Ann Thorac Surg.

[11] Lizhong S, RuiDong Q, JunMing Z, YongMin L, Qian C, Jun Z (2011). Repair of Acute Type A Dissection: Our Experiences and Results. Ann Thorac Surg.

[12] Dipros P, Herbertson MJ, O'Shaughnessy D, Gill RS (2005). Activated recombinant factor VII after cardiopulmonary bypass reduces allogeneic transfusion in complex non-coronary cardiac surgery: Randomized double-blind placebo-controlled pilot study. Br J Anaesth.

[13] Welsby J, Monroe DM, Lawson JH, Hoffmann M (2005). Recombinant activated factor VII and the anaethetist. Anaesthesia.

[14] Willis C, Bird R, Mullany D, Cameron P, Philisps L (2010). Use of rFVIIa for critical bleeding in cardiac surgery: dose variation and patient outcomes. Vox Sang.

[15] Nicholas AD, Bhattacharya SD, Williams JB, Fosbol EL, Lockhart EL, Patel MB, Gaca JG, Welsby IJ, Hughes GC (2012). Intraoperative use of low-dose recombinant activated factor VII during thoracic aortic operations. Ann Thorac Surg.

[16] Hendrisks HG, Meijer K, De Wolf JT, Porte RJ, Klompmaker IJ, Lip H, Slooff MJ, Van der Meer J (2002). Effects of recombinant activated factor VII on coagulation measured by thromboelastography in liver tansplatation. Blood Coagul Fibrinolysis.

[17] Ponschab M, Landoni G, Biondi-Zoccai G, Bignami E, Frati E, Nicolotti D, Monaco F, Pappalardo F, Zangrillo A (2011). Recombinant activated factor VII increases stroke in cardiac surgery: A meta-analysis. J Cardiothorac Vasc Anesth.

[18] Butensa S, Brummel KE, Branda RF, Paradis SG, Mann KG (2002). Mechanism of factor VIIA-dependent coagulation in hemophilia blood. Blood.

[19] Elsherbiny IA, Shoukry A, Tahlawi MAE (2012). Mean platelet volume and its relation to insulin resistance in non-diabetic patients with slow coronary flow. J Cardiol.

[20] Ernofsson M, Thelin S, Siegbahn A (1997). Monocyte tissue factor expression, cell activation, and thrombin formation during cardiopulmonary bypass: a clinical study. J Thorac Cardiovasc Surg.

[21] Dietrich W, Spannagl M (2002). Caveat against the use of activated recombinant factor VII for intractable bleeding in cardiac surgery. Anesth Analg.

[22] Gill R, Herbertson M, Vuylsteke A, Olsen PS, Von Heymann C, Mythen M, Sellke F, Booth F, Schmidt TA (2009). Safety and efficacy of recombinant activated factor VII a randomized placebo-controlled trial in the setting of bleeding after cardiac surgery. Circulation.

[23] Mitra B, Phillips I, Cameron PA, Billah B, Reid C (2010). The safety of recombinant factor VIIa in cardiac surgery. Anaesh Inensive Care.

[24] O'Connell KA, Wood JJ, Wise RP, Lozier JN, Braun MM (2006). Thromboembolic adverse events after use of recombinant human coagulation factor VIIa. JAMA.

[25] Johnson SJ, Ross MB, Moores KG (2007). Dosing factor VIIa(recombinant) in nonhemophiliac patients with bleeding after cardiac surgery. Am J Health Syst Pharm.

