# Supplementary figures and images for: Combination use of platelets and recombinant activated factor VII for increased hemostasis during acute type a dissection operations
Source: J Cardiothorac Surg. 2014 Sep 2;9:156. doi: 10.1186/s13019-014-0156-y (PMC4156631; doi:10.1186/s13019-014-0156-y)

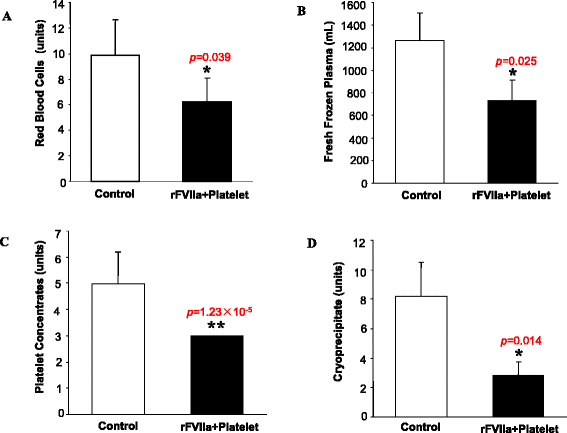

Supplement: Supplementary file 1 — Authors’ original file for figure 1 [file 13019_2014_156_MOESM1_ESM.gif]

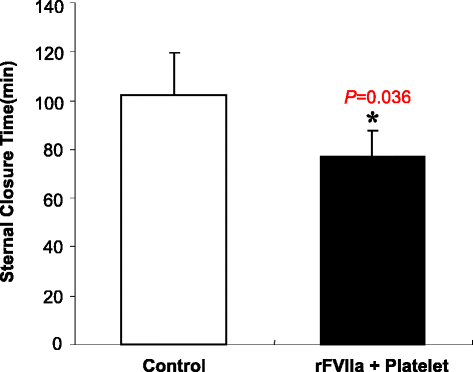

Supplement: Supplementary file 2 — Authors’ original file for figure 2 [file 13019_2014_156_MOESM2_ESM.gif]
